# Integrating Community Health System Activities and Participation into the Primary Healthcare System for Ensuring Effective Community Participation and Achieving Health Goals in Nigeria

**DOI:** 10.5334/aogh.5174

**Published:** 2026-09-18

**Authors:** Enyi Etiaba, Chinelo Obi, Obinna Onwujekwe

**Affiliations:** 1 Health Policy Research Group, University of Nigeria, Enugu Campus, Enugu, Nigeria; 2 Department of Health Administration and Management, University of Nigeria, Enugu Campus, Enugu, Nigeria

**Keywords:** community-led activities, household, PHC system, integration, social determinants of health

## Abstract

*Introduction:* In Nigeria, communities have long organised health and social actions that address local needs but remain weakly integrated into the formal primary healthcare (PHC) system. Understanding these approaches and how they interface with PHC is important for advancing universal health coverage (UHC).

*Objective:* This study examined community-driven health and social actions and explored pathways for their integration into the formal PHC system.

*Method:* A qualitative study was conducted in three purposively selected Nigerian states: Anambra, Akwa Ibom, and Kano. Data were collected through face-to-face in-depth interviews with 11 formal healthcare providers, 31 informal healthcare providers, and 19 traditional and religious leaders, and 12 focus group discussions with community members. Data were analysed deductively using the Expanded Health System Building Blocks and the World Health Organization Operational Framework for Primary Health Care.

*Results:* Communities across the study sites had established diverse, self-organised health and social actions that filled gaps in primary healthcare delivery. These included community-financed drug distribution and insurance schemes, subsidised care by informal providers, health education, sanitation, food support, and community safety initiatives. Led by local leadership structures, these activities were sustained through collective responsibility. Interfaces with the PHC system had emerged in several settings, including PHC-led training of traditional birth attendants and informal providers, revealing a community-generated layer of healthcare delivery with potential for systematic integration.

*Conclusion:* Our findings highlight opportunities to strengthen linkages between existing community initiatives and PHC governance, financing, and accountability structures to better harness community contributions to health and its social determinants and support progress towards UHC.

## Introduction

Households and community-generated activities that produce or improve their health provide a platform that can be capitalised on to strengthen primary healthcare (PHC) and help countries to achieve universal health coverage (UHC). The majority of these community-based activities are contextualised approaches to health promotion and disease prevention, addressing health and social determinants of health (SDH) [1, 2]. The commission on SDH recognises individuals (households) and communities as the micro- and meso-level entry points, respectively, for addressing SDH [3].

Globally, communities have proven to be vital assets for addressing SDH through collaborative, locally driven approaches [4]. Some programmes that had the participation of some politicians, philanthropists, and community members were viewed as legitimate, credible, and relevant, and integration of these programmes within the existing health systems enhanced programme compatibility with the health systems’ governance, financing, and training functions [5, 6].

While some countries report substantial health gains from community engagement, others show more modest effects [7]. Bottom-up community activities have reduced the occurrence of non-communicable diseases in low-and-middle income countries like India and Indonesia [8].These activities have also increased the sense of ownership and responsibility, use of indigenous knowledge, and reduction in cost [9].

Bottom-up production of health by households and communities has long been recognised in the African region [3]. People-centred bottom-up health-related activities can complement existing top-down approaches to further strengthen the health system towards achieving UHC. Engaging community members can help extend the reach and acceptability of healthcare services, including among underserved populations, and contribute to a more responsive health system [10].

In Nigeria, the contribution of communities to health is particularly important given the country’s substantial and changing disease burden. Communicable, maternal, neonatal, and nutritional diseases remain predominant, accounting for about 65% of deaths in 2019, compared with 29% from non-communicable diseases [11]. Malaria and neonatal disorders were the two leading causes of years of life lost, while non-communicable diseases are becoming increasingly important [11]. This combination of health problems creates considerable health needs, many of which are also influenced by the social, economic, and environmental conditions in which people live.

Recent evidence in Nigeria shows various linkages and horizontal collaborations between communities and their PHC facilities, some of which are community-led whilst others are PHC health facility-led [12, 13]. This paper further explores community-led, otherwise referred to as organic or community organising activities. It moves beyond collaboration to integration. It highlights community-initiated contextual approaches to health that have contributed to improved community health and explores if and how these activities have been or can be integrated into the PHC system to further strengthen healthcare provision at the community level, towards achieving UHC.

Community members and community-based organisations can effectively identify health priorities, address health concerns, manage financial and personal processes locally, and evaluate and hold health systems accountable. In addition, the utilisation of community members or organisations as health workers and promoters to deliver basic health services has been identified as a strategic approach to mitigate the growing shortage of healthcare workers.

Community organisations help identify health priorities, manage local resources, and hold health systems accountable [1, 2, 12]. They also act as trusted intermediaries who translate health messages into culturally relevant forms and extend care to underserved groups [8].

Community members’ knowledge of their local context can support the adaptation of health services to local needs and extend the reach of the health system. By leveraging these capabilities, community-based approaches may facilitate wider engagement, improve health knowledge, and help address barriers to care [14].

Engaging community members in addressing their health issues nurtures a sense of ownership, which can potentially facilitate the integration of community healthcare interventions [14]. This can be crucial for guiding behavioural modification interventions and facilitating adaptation to changing environmental, demographic, and epidemiological conditions [15].

Community actions and initiatives for health are implicitly encapsulated under the community health system (CHS) umbrella. However, debates persist about how to define CHS, determine the optimal roles for communities in formal service delivery [16, 17], or establish criteria for optimal participation of communities in formal health service delivery [18]. In Nigeria, however, CHS remain largely informal and ungoverned [2].

This paper contributes new knowledge on the various forms of community participation in health. It further demonstrates how household- and community-driven efforts that address health needs and local determinants can be meaningfully integrated into the formal PHC system. It provides policy and strategic options that may inform PHC planning and broader health system strengthening efforts.

## Methods

### Study setting and design

Our qualitative study was undertaken in three purposively selected states across three geopolitical zones: Anambra State in the southeast, Akwa Ibom State in the south-south, and Kano State in the northwest. The states were selected to capture contextual diversity, while also considering the research team’s prior research experience in these settings. Nigeria has 36 states and a federal capital territory, organised into six geopolitical zones. Each state is further divided into local government areas (LGAs), wards, and communities.

#### Contextual characteristics

Akwa Ibom State is the highest oil and gas producing state. Despite a high literacy rate (males 75.5%; females 80.6%) [20], a strong belief in witchcraft remains, which affects health-seeking behaviour and shapes perceptions of health security [19]. Kano State still has a high rate (80.8%) of obstetric deliveries outside the health facility, and a very low female literacy rate (37.8%) compared to males (71.3%) [20]. Anambra State has the highest literacy rate of the three study states (males 89.1% and females 87%) and a high rate (90.4%) of facility-based obstetric deliveries [20].

Healthcare across the three states is provided through primary, secondary, and tertiary facilities. At the PHC level, nurses, midwives, community health officers (CHOs), and community health extension workers (CHEWs) deliver services, although shortages and uneven distribution of these health workers remain a challenge. In Akwa Ibom, an assessment of PHC facilities found that none of the facilities studied met the recommended minimum staffing requirements, with key health workers unevenly distributed across facilities [21]. Similar workforce shortages have been reported in Kano State, where only 467 nurses and midwives were available against an estimated requirement of 3,082, alongside shortages in other key PHC cadres [22]. In Anambra, health workforce shortages have also been reported, with 82% of PHC facilities assessed in one study operating with less than 20% of the nationally recommended number of health workers [23]. These differences in the availability and organisation of formal healthcare are important to understanding how communities respond to local health needs and interact with PHC services.

### Data collection

Data were collected through 61 face-to-face in-depth interviews (IDIs) and 12 focus group discussions (FGDs). The IDIs included formal healthcare providers, informal healthcare providers (IHPs), and traditional and religious/community leaders, while FGDs were conducted with community members, comprising approximately 7–10 participants per group. The distribution of IDIs and FGDs across participant groups is presented in Table 1.

**Table 1:** Study participants and data collection methods.

PARTICIPANT GROUP	DATA COLLECTION METHOD	NUMBER OF IDIs/FGDs
Formal healthcare providers	In-depth interview (IDI)	11
Informal healthcare providers	In-depth interview (IDI)	31
Traditional and religious/community leaders	In-depth interview (IDI)	19
Community members	Focus group discussion (FGD)	12 FGDs
**Total**		**61 IDIs; 12 FGDs**

Interview questions addressed approaches they take to ensure proper health-seeking and the enhancement of healthcare for community members. Interview sessions were audio-recorded, and field notes were taken following informed consent from participants. Interviews were conducted in English and/or the predominant local language in each state: Ibibio in Akwa Ibom, Igbo in Anambra, and Hausa in Kano. Research assistants who were resident in the study states and fluent in English and the respective local languages were trained and included in the data collection team. Data collection was conducted between August and October 2022.

### Data analysis

Interviews conducted in English were transcribed verbatim, while interviews conducted in local languages were translated into English and transcribed by research assistants fluent in the respective languages. Deductive themes derived from the Expanded Health System (EHS) building blocks framework were initially used to develop the codebook. Transcripts were then manually coded and analysed using relevant themes from both guiding frameworks. Themes relevant to this study included community and household activities and health system building blocks, household production of health and SDH, and societal partnerships. For quality assurance, four transcripts were independently coded by members of the research team, followed by team meetings to reconcile differences and finalise the codebook. All transcripts were subsequently coded and organised using a Microsoft Excel template.

### Analytical framework

**Figure 1:**
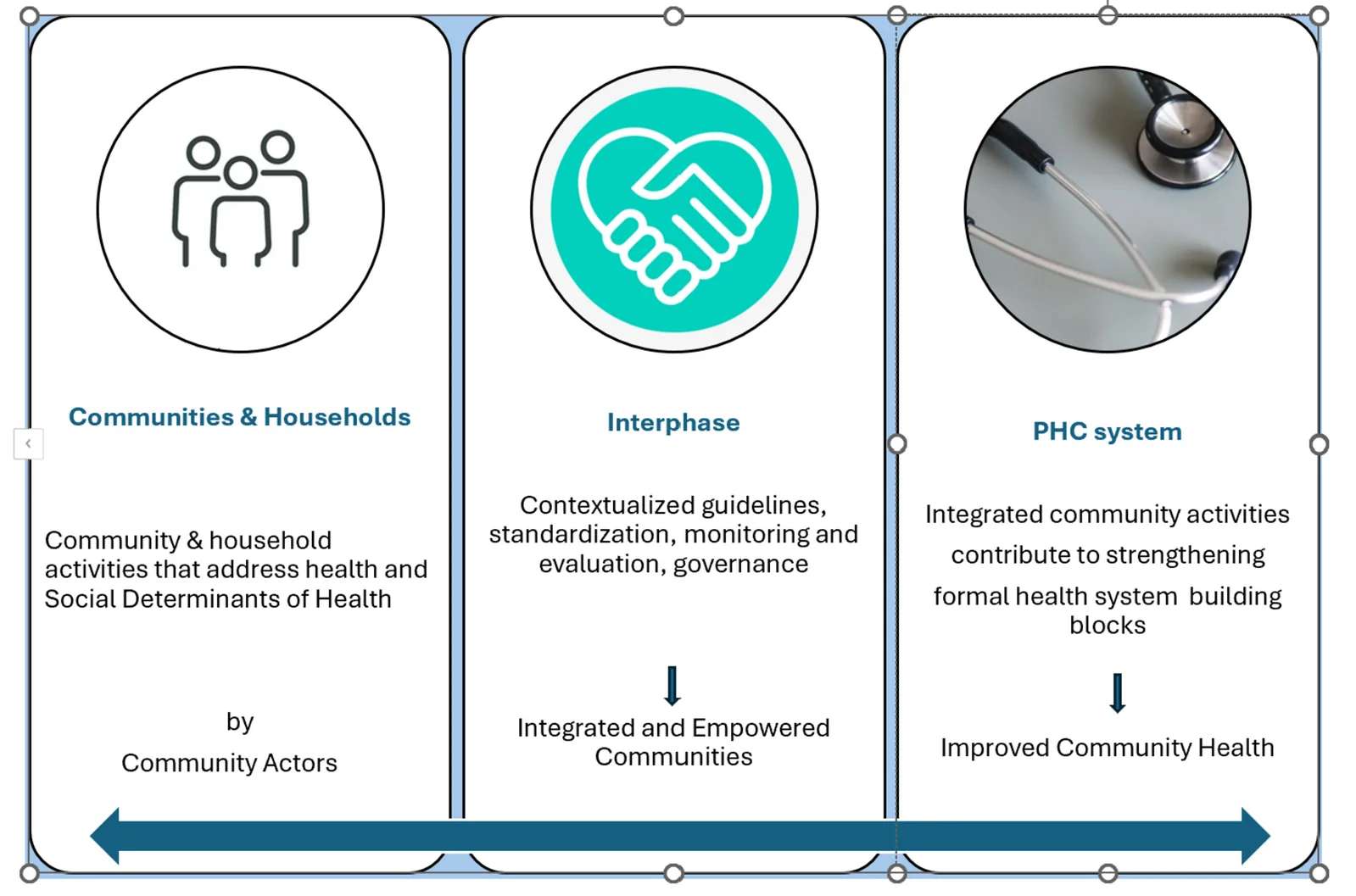
Study analytical framework (adapted from Sacks et al. and WHO Operational Framework for PHC).

Our analytical framework (Figure 1) depicts the interface where community- and household-led actions connect with the formal PHC system through locally developed practices and accountability mechanisms. This interface is not a separate structure, but a dynamic space of integration where collaboration and community empowerment strengthen PHC delivery.

## Ethical Consideration

This study was conducted in accordance with the ethical principles outlined in the Declaration of Helsinki. Ethical approval was obtained from the University of Nigeria Teaching Hospital, with reference number: NHREC/05/01/2008B-FWA00002458-1RB00002323. The study was conducted in accordance with the local legislation and institutional requirements. The participants provided their written informed consent to participate in this study.

## Results

The findings are presented in three sections. The first section presents identified community activities that address health and SDHs. The second section presents pathways for integrating these activities into PHC systems, and the third section examines factors that may facilitate or constrain integration.

### Community activities that address health

The study identified three categories of community-led activities that directly addressed health needs: (i) distribution of free drugs and medical supplies; (ii) health screening, education, and promotion; and (iii) community-based health insurance.

#### Distribution of free drugs and medical supplies

In some communities, community members pay patent medicine vendors (PMVs) for certain medications (e.g., Vitamin A and antimalarial drugs) to ensure that those who cannot afford them receive them for free. Some private individuals also give out medical supplies to these vendors to share freely with their customers. There are also community PMVs who subsidise or offer free treatment to community members on occasion:

*There are people who are sick; when they come to me and they don’t have money, I treat them for free. I sell drugs at a cheap rate, and during the Christmas period, I give my customers free drugs. I am also planning to buy a BP apparatus and blood sugar testing kits to test people for free (IDI, PMV, Anambra).*

Some communities in Kano and Anambra States organised periodic health outreaches involving medical personnel, community volunteers, and health workers, providing free medicines, healthcare, and referrals. Although largely philanthropic, some activities already involved formal health providers and referral linkages. In Kano, one such activity was an annual outreach organised by a youth forum around Christmas.

*… Community-oriented health programme, the one I know and can say is usually done annually is the one the youth forum normally organises towards Christmas… they will bring a doctor, then drugs are given to sick patients. The people with serious issues will be referred to the hospital, then assistance will be sought on behalf of the person if the person cannot foot the bill. Assistance involved approaching community members who could contribute towards the cost of care (FGD, community member, Kano).*

#### Health screening, health promotion, and education

Free health checks are organised by community elites for early diagnosis of ailments and to identify those who need care. Those in need of treatment are booked for care, and their bills are taken care of. These community-based interventions are driven by indigenes of the respective communities and usually organised to take place annually at about the same time of the year, but other stakeholders are also involved. A formal healthcare provider in Anambra State reflected:

*“* *Yes, when I newly came here …this is the man that welcomed me in this town. He is a doctor too at the eye hospital. The condition he gave me is that I must do a free medical check, which I did at the town hall. I did it myself. I did a free lab test for everybody for two weeks...”(IDI, med lab scientist, Anambra).*

Health promotion and health education were enabled in these communities by leveraging various actions and activities. Town criers and outreaches were among the methods leveraged to create health and healthcare awareness. They were also used as a means to prevent infections, raise awareness about diseases, and promote health services available to the community.

*… what the committee, in conjunction with the community leaders, do is, they make sure they create awareness to the members of the community by using town criers, by talking to people one on one on the benefits of health and to stay healthy…. there are people that are not well informed in the community. When the town crier makes announcements there begins to be vaccination… (FGD, community member, Akwa-Ibom).*

Religious leaders and school leaders, who are highly respected in the communities, also got involved in health education, promotion, and sensitisation. Community leaders in Kano also took actions to stop the spread of diseases in their community:

*When there was an outbreak of cholera, one of us took action when they saw it, they quickly told the household that they have to go to the hospital. Through that they got our people to come to the hospital. When they come to the hospital, they will bring all their household so that it can’t be spread to anyone. That’s how we use to do it (IDI, WDC chairman, Kano)*

The community leaders in Akwa-Ibom also organised seminars on sex education to educate the populace. There were also initiatives implemented during the COVID-19 pandemic in Akwa Ibom State to speak to the people on the benefits of healthcare.

#### Community-based health insurance

Community health insurance was established as a way to fund healthcare, with healthy community members contributing towards the care of those who were ill.

*Our people started what they called community health insurance and health financing scheme... They came and started a community healthcare and community financing scheme that enables the healthy ones to finance the health of those that are not healthy. As a result, if you pay one hundred Naira premium (100) monthly dues of one thousand two hundred Naira only (#1,200.00) per year, you will be entitled to enter the scheme with a card. So, they use the three health centres in Igboukwu as their base. Our people donate to finance (IDI, Director, Anambra).*

This scheme was adopted by some other communities, and when the state health insurance was introduced, the community schemes were absorbed into the state scheme, and philanthropists continued to pay premiums for the less well-off in their communities.

*For instance, when O.(the governor) introduced ASHIA (Anambra State Health Insurance Agency), it went out of the community health financing scheme and governments now dominate. I will show you the cards for the local scheme. He funds it on his own as a way of giving back to the community. All these are delivered by philanthropic acts (IDI, community member, Anambra).*

### Community activities that address social determinants of health

#### Provision of security

In most of the PHCs, the security guards were sponsored by community members. They were involved in appointing a security guard, usually a member of the community and also pay them a stipend. In one study state, this reportedly improved access to and utilisation of PHC services, as reflected by this respondent:

*…in my area, there is a hospital where armed robbers always disturb them, but since we started working, all these have reduced drastically, and the staff can now go to work without fear and patients can easily visit the hospital for treatment any time. Also, when they (facility staff) go for programs to share medications and other medical products, we also try to ensure their protection so that these things do not get snatched or people interrupt or disturb them (FGD, security guard, Anambra).*

*The WDC in our community provide physical support, they provide security to the PHCs when there are health programs being carried out in the facility (IDI, OIC, Akwa-Ibom)*

#### Environmental sanitation

Enforcement of environmental sanitation and hygiene was the most common form of community activity addressing SDH. Community leaders mobilised their members to engage in sanitation, cleaning drains and their surroundings. This is mainly championed by the village heads and carried out by the youths. As one community member reflected:

*“There’s what we call community sanitation in our community; I was part of it almost seven months ago in our area. They organized it under our village head at that time. They bring us out saying that those who are selling akara [beancake] in the street near the gutters should stop selling there, including the cooking and selling of food along the gutter” (FGD, Trader, Kano).*

Community leaders took responsibility for ensuring that people adhered to environmental sanitation regulations in their communities;

*We speak to them on the importance of seeing that the environment they live in is clean....In those days you know we use to have several diseases, they were thinking they were witches or their enemies, but now, they have realized that they are the architects of their own misfortune (IDI, village head, Akwa Ibom).*

#### Community food empowerment

Groups of farmers in communities in Anambra State coalesced and produced a plan for enhanced food production. Although encouraged and eventually financially supported by the government, the communities initiated the design of contextually relevant plans to enhance food production during the COVID-19 pandemic. A government stakeholder reflected:

*It’s just for the poor members of the community. They have a community facilitator who gets information and guides them to meet up with the requirements…they form a cooperative of 40 – 100 persons…they organize themselves and once they meet the requirements through the community facilitator, they come forward for approval…..we can’t say that we have completely eradicated poverty, but I must say that we are proud of the results for now…..there is an increase in the number of poor people accessing the inputs. We now have two thousand, seven hundred and seventeen (2,717) of them scattered across fifteen local governments in Anambra State...the increased food security in the state contributed to increased yield…we can say that most of them may be feeding better than before, which may have in turn strengthened their health status. (Officer, Agriculture programme).*

## Strategies

These activities were initiated, organised, and sustained by various community members (youths, women’s associations, men’s associations, village heads, and other leaders) and philanthropists (politicians and private individuals). Community philanthropy was a key strategy. In Anambra and Kano states, wealthy community members are known to contribute to community health in various ways, either by contributing medicines and food, or sponsoring medical outreach programmes:

*Someone in the community, the philanthropies who, now have the will to help the community in terms of their health and wellbeing. He will now gather a program or sponsor a program, a health program that will now benefit the community… The eye program is about screening the community in search of those with eye problems. Some will be screened; if they have simple glaucoma, a drug will now be prescribed for them. Some may be prescribed reading glasses to improve their sight... (IDI, formal provider, Kano)*

In some communities in Anambra State, some of the philanthropists in the communities enrol some community members in the state insurance scheme and take care of their premiums.

*Some politicians enlisted the poor people in the community under this ASHIA (the state insurance), so my family and I come here anytime we have health challenges, and we only spend little money (FGD, Trader, Anambra).*

An emergent finding was that philanthropy is not prominent in one of the study states, Akwa Ibom, where affluent members of the community do not ‘give back’ out of fear of being attacked by witchcraft. This finding reinforces the need for contextualised approaches.

### Potential pathways for integration of contextualised community-initiated activities into the formal PHC system

Following community participation and activities to improve healthcare, some formal providers initiated steps to foster community involvement. Evidence of harmonisation of these contextualised community activities included training programmes for informal providers and community members, recruitment of certain community members to assist during formal health activities, and creation ofn accountability structures for managing contributions from community philanthropists.

#### Training of informal providers and community leaders by formal providers

Training and retraining were provided to IHPs, including TBAs and PMVs, although the content varied by provider group. TBAs described training on delivery practices, recognition, and management of complications, prevention of communicable diseases, and appropriate referral to health facilities. Training reported by PMVs covered malaria, family planning, pneumonia, and patient management. Community intermediaries, including TBAs, also received training on household health education and community mobilisation.

*Yes, and we’re really enjoying the retraining because the training is encouraging us, and like before most of the women when they give birth at home they don’t care to go to the hospital but now as a result of our work they do come to the hospital, and all happens as a result of the training, and all these are among our work, and now even giving birth at home is very rare and even now that we are talking there is a woman in labour room that is about to give birth (FGD, TBA, Kano).*

Participants reported that training and retraining were provided by both government and non-governmental organisations, although the frequency varied. Non-governmental organisations were described as providing retraining more frequently in some settings, while trainees also received support for transportation and food during training. One participant explained:

*Sometimes before the government calls us for a single retraining, the NGO has trained us more than 2 to 3 times. They give us money for transportation and food during the training*… (IDI, PPMV, Kano).

Formal providers also included health talks for the community in their work schedule. They organised seminars at scheduled times for the community members in the health facility. Participants reported that these sessions increased their knowledge and informed the advice they gave to other community members about seeking care.

*There was a seminar at the health center that required women to attend, so we were taught and advised on these things to do, and that was how I learned and knew them. So, I advise and encourage other women to check their blood pressure. I used to check my blood pressure on my own before the seminar, so it was additional knowledge for me. I advise women, especially those who go to markets or farms and are stressed after a long day’s work, to check their blood pressure frequently because stress causes an increase in blood pressure, which most women don’t realize and instead believe is witchcraft (IDI, Woman leader, Akwa-Ibom).*

#### Recruitment of community help during outreaches

There are assigned days for outreaches in every community, and in communities with few formal providers and CHEWs, community members were recruited to assist during outreaches. The roles of these recruited community members were not to vaccinate or perform healthcare duties but rather to support the outreach activities.

*The OIC of each ward knows the people that she is working with in each of the communities; there are people in the community whom their leaders know can do the job well. Like crowd control and community mobilization. Yes, there are criteria because a village woman can’t be a vaccinator; the vaccinator must be a trained nurse or CHEW. The village people will be recruited as town criers, house-to-house mobilizers, or crowd control people. We recruit them according to their abilities (IDI, Civil Servant, Anambra).*

#### Accountability structure for community philanthropy

Healthcare facilities in some communities established structures to foster and ensure accountability for funds donated by community philanthropists.

*In every organization there is a leader. So, any donor will go through the hospital health committee leader and the two of them will come together and give the description of what he is giving to the hospital (FGD, Community member, Kano)*

Another community had an environmental and health unit embedded in the formal health facility and tasked with health promotion. The community had also activated a yellow fever campaign to vaccinate community members.

*Okay, currently the community is participating in this yellow fever because now we are, we are going to activate this yellow fever program in the state. So, the community are being selected so that those we want to participate fully… because most of the oldest (members of the team) are from the community. We use our health workers as vaccinators and the oldest who are from the community to encourage community members to participate fully in the yellow fever vaccination. Yes. The in-charge of the facility is the vaccinator… vaccinator of that area because people know she is in charge of the committee (IDI, Primary Health Care Coordinator, Kano).*

### Facilitators for integration

Integration of community-organised initiatives into the PHC system was supported by strong leadership, effective coordination mechanisms, philanthropy, collaboration with health workers, and inclusive community engagement. Structural mechanisms such as the active presence of Ward Development Committees (WDCs) provided a foundation for organising and coordinating community health activities. As one officer explained:

*The presence of the WDC is a key enabler to community engagement and participation; they are always there… anytime you call them, they render their support (IDI, OIC, Akwa Ibom).*

Another respondent emphasised:

*…they have leaders, and their leaders monitor the activities they are involved in (IDI, Civil Servant, Anambra).*

Procedural supports, including agency-led sensitisation and technical guidance, provided communities with knowledge and support for their activities. A civil servant described how PHC workers were invited to deliver health education during community initiatives, explaining that,

*…when there is a new community-led program, they do invite us and we go and give them health talks and sensitize them about their health. Like last year, there’s a philanthropist from Mbaukwu that was organizing a widows’ group and she asked us to come and give them health education (IDI, Civil Servant, Anambra).*

Community leadership and philanthropy further strengthened integration. Across the study states, influential individuals and political leaders sponsored health outreaches and provided financial support to vulnerable households, bridging service gaps and linking community efforts to PHC systems. As one provider explained:

*…someone in the community, the philanthropists who have the will to help, will sponsor a health programme that benefits everyone. For example, the eye programme screened the community and helped those with problems (IDI, Formal Provider, Kano).*

A participant similarly noted:

*Some politicians enlisted the poor people in the community under ASHIA, so my family and I come here anytime we have health challenges, and we only spend little money (FGD, Trader, Anambra).*

Collaboration and mutual trust between community members and PHC workers also enhanced integration. Health workers were often invited to supervise or participate in community activities, providing opportunities for coordination between community initiatives and formal health services.

*When we organize our health outreach, we invite the nurse from the health centre; she helps to check blood pressure and give advice on what to do (FGD, Community Member, Kano)*

Training and engagement of informal providers, such as traditional birth attendants and PMVs, by PHC staff provided another important link. Participants reported that these sessions helped informal providers better understand when referral to formal services was appropriate.

*The training made it easier for us to know when to refer people; before, some of us were treating everything, but now we know where our work stops (IDI, Informal Provider, Akwa Ibom)*

Inclusive decision-making and participation of diverse community groups—men, women, youths, and vulnerable populations—also facilitated successful integration. A local leader explained:

All the community groups are meant to be involved in the community discussion—the men, the women, the youths, the vulnerable. After that, we embark on voting and the need is chosen. If there is a similar project in a neighbouring community, then we guide them to choose another project *(IDI, Head of CSDP, Akwa Ibom).*

### Constraints

While communities demonstrated strong commitment to organising and sustaining health and social activities, several factors constrained their integration into the formal PHC system. Limited financial and material resources frequently disrupted continuity of activities, as community members struggled to mobilise sufficient support to complete projects.

*We the women in this community have also started the foundation of our health post, but it has been at the foundation level. The men are not helping us (FGD, Housewife, Anambra)*

*We planned to buy materials and continue, but the money was not enough, so it stopped there (IDI, Community Leader, Akwa Ibom)*

Gender and social dynamics also influenced participation and decision-making, often excluding women or limiting their roles in community projects.

*If women bring up ideas, the men will not always agree. They say it is not their duty to do those things (FGD, Women’s Group, Kano)*

Weak institutional linkages between PHC facilities and community groups hindered sustained collaboration. Health workers were not consistently engaged in supervising or supporting community-led efforts.

*Sometimes we invite them [health workers], they promise to come and supervise what we are doing, but they don’t show up (FGD, Community Member, Akwa Ibom)*

*We need government to recognize what we are doing; if not, we will continue on our own, and it may not last (IDI, Community Volunteer, Anambra)*

Participation was further constrained by the exclusion of certain groups, such as youths and low-income households, from decision-making, which weakened collective ownership.

*Only a few people decide what to do. Sometimes we hear about it when everything is already done (FGD, Youth, Kano)*

Finally, informal funding and contribution systems often lacked clear accountability, discouraging sustained engagement.

*People will contribute money, and at the end, no one will account for how it was used. That discourages others (FGD, Community Member, Anambra)*

These findings reveal that while communities possess strong internal motivation to act for health, sustained impact requires deliberate policies that formalise their contributions, strengthen accountability, and link community efforts to PHC governance and financing systems. Across the study states, communities are actively shaping health through community-organised initiatives that include free drug distribution, community-based insurance, sanitation, food support, and security for PHC facilities. These actions reflect strong social cohesion and informal governance that sustain local well-being. Integration with formal PHC structures is gradually emerging through training of informal providers, engagement of community volunteers, and accountability for philanthropic support, driven by local leadership and WDCs. Yet, limited financing, gender norms, and weak institutional linkages threaten sustainability. These findings highlight that communities are already functioning as partners in health delivery, underscoring the need to suggest opportunities to strengthen these linkages within PHC systems and potentially support their continuity.

## Discussion

This study identified organic, bottom-up community activities that contribute to healthcare delivery and address health and its social determinants. It also identified existing linkages between some community activities and the formal PHC system, as well as opportunities to strengthen and sustain community participation in PHC. The study also identified actions undertaken to integrate household and community activities, implicitly within the ambit of the CHS, into the PHC system, as well as opportunities to enhance, harness, sustain, and scale up systematic community participation in PHC.

This study provides new insights into how communities contribute to PHC by drawing evidence from diverse sociocultural and governance contexts in Nigeria. Its strength lies in the depth and breadth of perspectives captured, combining the voices of formal and informal providers, community and religious leaders, and service users to reveal how locally organised efforts complement and interact with the PHC system. Guided by the Expanded Health System Building Blocks and the WHO–UNICEF Operational Framework for PHC, the analysis offered a coherent lens for understanding the processes, enablers, and constraints that shape integration at the community level.

The health promotion activities identified in this study highlight the role that community participation can play in shaping health knowledge and health-related practices [8]. Activities such as health education, encouragement of appropriate care-seeking, and promotion of physical activity demonstrate how community action can complement formal healthcare by addressing behaviours and practices relevant to disease prevention and management [4]. Similar community-based health promotion activities have been reported in Kenya [24].

Community-based health insurance, identified in this study as one mechanism for integrating community activities into the formal health system, has been reported to increase healthcare utilisation in various studies [25, 26]. Though it has been reported to reduce financial barriers to access but does not effectively protect the enrollees [25]. Another study found the overall effectiveness of community based health insurance in improving healthcare utilisation to be satisfactory, although the utilisation rate among community members remained below the programme target and the WHO recommendation [26].

Training of IHPs has been reported to improve the management of disease conditions in communities. An Indian study found that training IHPs improved appropriate case management, although it did not reduce the use of non-essential medicine [27]. A tuberculosis (TB) management study reported that non-medical professionals can handle the management of TB cases with appropriate training. It also highlighted that the involvement of IHPs with the right training could improve healthcare outcomes [28]. A study in India highlighted a notable knowledge gap between trained and untrained IHPs [25].

The importance of community involvement in environmental sanitation and personal hygiene was also reported in a Tanzanian study. The study emphasised that community involvement and awareness were the major reasons for improving environmental health. Ensuring sustainability of this venture in any community, the members must be involved and central in the process [29]. A Nigerian study reported excessive dependence on the government to provide environmental sanitation infrastructure, which showed that communities did not play the leading role in the area of environmental sanitation. The study recommended greater community involvement in this area to reduce overdependence on the government, which hinders the provision of such infrastructure [30].

Community members volunteering as security guards at the PHC centres was consistent with a South African study that reported on community activities that influenced the PHC centres in various communities. The study reported that community members who were part of health committees assisted with the day-to-day running of health centres. One of the activities they were involved in was fto serv as security guards. Other roles included cleaning the facilities and working as receptionists. They also acted as mediators when patients expressed dissatisfaction [30].

Although the community food empowerment programme did not directly involve the health sector or PHC system, it highlights a crucial community-based approach to addressing SDH. Similar community-based approaches have been used to address social needs through collaboration between primary care and community organisations [31], while community-based self-management interventions have demonstrated improvements in several health and well-being outcomes [32].

The study was conducted in three states and may therefore not capture the full range of regional variations across Nigeria’s diverse sociocultural contexts. As with most qualitative studies, participants’ views were shaped by context and perception, and social desirability bias may have influenced some responses. Nonetheless, triangulation of data sources, inclusion of multiple stakeholder groups, and iterative analytical validation enhanced the credibility and trustworthiness of findings. The cross-sectional design also limited the ability to observe changes in integration over time, highlighting the need for longitudinal studies to explore sustainability and policy adoption.

In conclusion, our findings show that contextually relevant, bottom-up community activities can interface with and, in some settings, be integrated into the formal PHC system. TPolicies are needed to support the integration and sustainability of these contextually relevant community activities. Existing organisational and monitoring structures can also be leveraged to strengthen accountability.

## Data Availability

The datasets used and/or analysed during the current study are available from the corresponding author on reasonable request.
